# Association between autoimmune thyroiditis and BRAFV600E/TERT promoter mutations in patients with papillary thyroid carcinoma from Central Asia, Kazakhstan

**DOI:** 10.1371/journal.pone.0351960

**Published:** 2026-07-20

**Authors:** Saya Kaidarova, Zhanna Mussazhanova, Zhanna Kozykenova, Hirokazu Kurohama, Akbota Targynova, Altay Dyussupov, Zhanar Yeleubayeva, Masahiro Nakashima

**Affiliations:** 1 Semey Medical University, Semey, Kazakhstan; 2 Nagasaki University, Atomic Bomb Disease Institute, Nagasaki, Japan; 3 Al-Farabi Kazakh National University, Almaty, Kazakhstan; 4 Kazakh Institute of Oncology and Radiology, Almaty, Kazakhstan; King Faisal Specialist Hospital and Research Center, SAUDI ARABIA

## Abstract

**Background:**

Autoimmune thyroiditis (AIT), including Hashimoto’s thyroiditis (HT) and Graves’ disease (GD), frequently coexists with papillary thyroid carcinoma (PTC). Chronic inflammation may influence thyroid carcinogenesis; however, the relationship between AIT and aggressive molecular alterations in PTC, particularly BRAFV600E and TERT promoter mutations, remains unclear. Data from iodine-deficient regions such as Kazakhstan are limited.

**Methods:**

We conducted a retrospective multicenter study of 231 patients with PTC from Kazakhstan treated between 2016 and 2020. Tumors were reclassified according to the World Health Organization 5th edition (2022). Clinicopathological characteristics, AIT status (HT and GD), BRAFV600E and TERT promoter mutation profiles, and proliferative activity (Ki-67) were analyzed. Associations between AIT, molecular alterations, and clinicopathological features were evaluated.

**Results:**

Most patients were female (83.1%) and <55 years (68.4%). AIT disease was present in 55.3% of cases, predominantly HT, while GD was uncommon (3.3%). BRAFV600E mutation alone was detected in 64.0% of patients, BRAF/TERT promoter co-mutation in 5.2%, TERT promoter mutation alone in 1.9%, and no mutations in 28.9%. TERT promoter-related mutations, particularly BRAF/TERT promoter co-mutations, were significantly more frequent in patients without AIT (p = 0.029) and were associated with lymph node metastasis, advanced tumor stage, older age, and larger tumor size. Patients with AIT had fewer aggressive molecular alterations. Patients with GD had higher Ki-67 labeling index and more aggressive clinicopathological features.

**Conclusions:**

Different AIT subtypes showed distinct molecular and clinicopathological patterns in PTC. HT tended to demonstrate fewer aggressive molecular features, whereas GD showed features suggestive of increased proliferative activity. A lower prevalence of TERT promoter-related mutations was observed in AIT-positive patients, suggesting possible differences in molecular aggressiveness. However, these findings require validation in larger cohorts with broader molecular profiling.

## Introduction

Recently, an increased incidence of autoimmune thyroiditis (AIT), including Hashimoto’s thyroiditis (HT) and Graves’ disease (GD), has been reported [1–3]. Inflammatory diseases cause chronic inflammation, which promotes tumorigenesis [4–7]. Although the association between chronic inflammation and cancer development is widely recognized, it may be underpinned by distinct pathophysiological mechanisms that drive cellular transformation and alter immune responses, thereby enhancing tumorigenesis.

Kazakhstan has historically exhibited areas of endemic iodine deficiency, with low iodine levels contributing to endemic goiter and other thyroid disorders across much of the country. Endemic iodine deficiency promotes thyroid dysfunction and may increase the prevalence of autoimmune thyroid disorders [8,9].

Papillary thyroid carcinoma (PTC) is the most common type of thyroid cancer, and is frequently encountered in patients with AIT [7,10,11]. Furthermore, several reports have suggested an association between thyroid carcinogenesis and AIT; however, the etiological association remains unclear [12–16]. Understanding this association is crucial for tailoring patient management and predicting patient outcomes. PTC has an excellent prognosis. Despite its indolent nature, distant metastasis occurs in 1–4% of patients and significantly worsens prognosis, reducing the 5-year survival rate to 28–53.3% [17].

HT frequently affects middle-aged female individuals and is 10 times more common in female individuals than in male individuals. Representative AIT causes hypothyroidism by damaging follicular cells via autoantibodies and is predominantly found in regions with adequate dietary iodine [18]. The proinflammatory state involved in the pathogenesis of autoimmune diseases is thought to be a risk factor for cancer development. As patients with HT have a 1.5-fold increased risk of incidental PTC, they should be counseled and periodically monitored for underlying thyroid malignancies [19]. GD is another common AIT disorder that causes hyperthyroidism through agonistic stimuli of the thyroid-stimulating hormone receptor (TR) by anti-TR antibodies, and occurs more than three times as frequently in female individuals as in male individuals. Although the association between GD and PTC has been extensively studied, it still remains controversial [19–28]. PTC coexists more frequently with GD, and PTC with GD is more aggressive than PTC in control participants [26,27]. Conversely, other studies have found a lower incidence of PTC in patients with GD, suggesting a potential suppressive effect of GD-related autoimmunity on thyroid cancer development. Moreover, PTC occurring with overt AIT is less aggressive than PTC in individuals without AIT [28].

The BRAFV600E mutation is the most prevalent driver mutation in PTC and critically influences tumorigenesis and disease progression. Mutations in the telomerase reverse transcriptase (TERT) promoter on chromosome 5 are significant genetic markers associated with adverse clinical outcomes in thyroid cancer [29–35]. These mutations create binding sites for transcription factors, notably enhancing telomerase activation by interacting with the GA-binding proteins transcription factor alpha and E-twenty-six 1 [30–33]. The presence of both BRAFV600E and TERT-p mutations correlates with increased tumor aggressiveness, recurrence, and mortality, and has been consistently associated with the most aggressive clinicopathological phenotype of PTC [36–40]. However, to the best of our knowledge, no combined study of BRAFV600E and TERT-p mutations in patients with PTC and AIT has been conducted.

In this study, we aimed to evaluate the association between AIT and BRAFV600E/TERT promoter mutations in patients with PTC from Kazakhstan.

## Materials and methods

### Participants

A total of 231 patients with PTC were included in this study. Thyroid tumor samples from the Kazakh patient population were collected at three prominent medical centers in Kazakhstan between 2016 and 2020: the Kazakh Institute of Oncology and Radiology in Almaty, the Center of Nuclear Medicine and Oncology in Semey, and the Multidisciplinary Center for Oncology and Surgery in Ust-Kamenogorsk. Notably, none of the patients had a history of radiation exposure. The tumors were reclassified according to the 5th edition of the World Health Organization’s Classification of Tumors of Endocrine Organs (2022) by three independent pathologists (M.N., H.K., and Z.M.).

### Mutation analysis

To analyze the mutation status, genomic DNA was extracted from formalin-fixed paraffin-embedded (FFPE) tissues using a Maxwell RSC DNA FFPE Kit (Promega, Madison, WI, USA), according to the manufacturer’s protocol. The largest tumor was used as a representative case of multifocal cancer. A guide slide stained with hematoxylin and eosin was used to detect the tumor areas. FFPE tissue sections were cut at 8 μm, and, depending on tumor size, 2–5 slides were prepared for further extraction. Tissues were deparaffinized in 80% xylene, washed twice with 100% ethanol, and centrifuged at 10,000 × g for 5 min at room temperature. The concentration of extracted DNA was measured using a NanoDrop ND-1000 spectrophotometer (NanoDrop Technologies, Wilmington, DE, USA).

### Droplet digital polymerase chain reaction analysis for TERT-p and BRAFV600E mutations

BRAFV600E and TERT promoter mutations (C228T and C250T) were analyzed using droplet digital PCR (ddPCR) with mutation-specific primer/probe assays (Bio-Rad, Hercules, CA, USA). Mutant and wild-type probes were labeled with FAM and HEX, respectively. Assay design and validation have been described previously [41–43].

Each 20-µL reaction contained ddPCR Supermix, 900 nM primers, 250 nM probes, and 5 µL DNA. Droplet generation, PCR amplification, and signal detection were performed using the QX200 ddPCR system (Bio-Rad) according to the manufacturer’s instructions. Data were analyzed using QuantaSoft software. Detailed assay conditions, including thermal cycling parameters and validation criteria followed previous studies [41,42].

### Statistical analyses

Statistical analysis was performed to identify the association between AIT and molecular status using the Chi-square test. Subgroup analysis was performed based on the distribution of patients with and without AIT, as well as HT and GD. Categorical variables were analyzed using the chi-squared test of association, whereas continuous variables were compared using the Mann–Whitney U test for two independent groups and the Kruskal–Wallis test for more than two independent groups. Some histopathological and molecular data were missing, meaning that mutation status and AIT classification were not available for all patients. Therefore, analyses were performed using an available-case (complete-case) approach depending on the variables included. As a result, sample sizes varied across analyses, and denominators differed between overall cohort analyses and subgroup analyses (e.g., mutation- and AIT-based analyses). Percentages were calculated based on the number of available cases for each variable. Given the exploratory nature of the study and the limited sample size in certain subgroups, no formal correction for multiple testing was applied. Reported p-values should therefore be interpreted with caution. Both parametric and nonparametric statistical tests, along with inferential statistical analyses, were performed using the IBM SPSS software (version 26) to analyze the entire dataset.

### Ethics approval

This study was conducted in accordance with the principles of the Declaration of Helsinki. This study was approved by the Local Ethics Committee of Semey Medical University, Kazakhstan (Protocol No. G-041.11.01.03-2021) and the Institutional Ethical Committee for Medical Research at Nagasaki University (#15062617-3).

## Results

### General clinicopathological characteristics of the patients

Table 1 summarizes the clinicopathological characteristics of the patients with PTC (n = 231). Among the 231 patients, most were female (83.1%) and aged <55 years (68.4%). Tumors >10 mm were observed in 85.7% of the cases, and lymph node metastasis was observed in 28.9% of the cases. Most patients had early-stage disease, with 54.8% classified as T1 and 90.9% as stage I. Distant metastasis was rare (0.5%). Autoimmune thyroid disease was present in 55.3% of patients, with varying degrees of HT, whereas GD was uncommon (3.3%). Molecular analysis showed BRAF mutations alone in 64.0% of the patients, BRAF/TERT-p co-mutations in 5.2%, TERT-p alone in 1.9%, and no mutations in 28.9%. Among patients with AIT, the majority had tumors > 10 mm, similar to those without AIT (p = 0.99). A comparable pattern was observed in patients with HT and GD, where most cases were associated with tumors >10 mm in size, indicating no significant association between autoimmune thyroid conditions and tumor size (S1 Table).

**Table 1:** General clinicopathological characteristics of patients with Kazakh PTC.

Characteristic		n/N (%)
**Sex**	Female	192/231 (83.1)
**Age group, years**	< 55	158/231 (68.4)
**Tumor size, mm**	≤10.0	34/231 (14.7)
**LN metastasis**	yes	57/197 (28.9)
**Pathologic T category**	Tx	2/197 (1)
	T1	108/197 (54.8)
	T2	58/197 (29.4)
	T3	28/197 (14.2)
	T4	1/197 (0.5)
**T1 vs. T234**	T1	108/195 (55.4)
	T2-4	87/195 (44.6)
**Distant metastasis**	yes	1/197 (0.5)
**Stage**	I	179/197 (90.9)
	II III IV	18/197 (9.1)
**AIT**	Yes	84/152 (55.3)
**HT**	No	73/152 (48.0)
	Mild	36/152 (23.7)
	Moderate	32/152 (21.1)
	Severe	11/152 (7.2)
**GD**	Yes	5/152 (3.3)
**Mutational status**	No mutation	61/211 (28.9)
	BRAF only	135/211 (64.0)
	*TERT*-p only	4/211 (1.9)
	BRAF/*TERT*-p	11/211 (5.2)

Kazakh PTC cases with a known status of Hashimoto’s thyroiditis and Graves’ disease are included in Table 1. PTC, papillary thyroid carcinoma; n, total number of cases; N, number of cases; mm, millimeters; LN, lymph node; T, tumor category, TNM classification (WHO 5th edition); Stage, I vs II + III + IV; AIT, autoimmune thyroiditis (Hashimoto’s thyroiditis or Graves’ disease); HT, Hashimoto’s thyroiditis; GD, Graves’ disease; no, control patients without AIT; BRAF, BRAFV600E mutation; BRAF-only, BRAFV600E without TERT-p; TERT-p-only, isolated TERT mutation; BRAF/TERT-p, double BRAFV600E and TERT-p mutation. Owing to missing data, denominators varied across variables. AIT-related analyses were performed only in cases with available histopathological data for AIT subtyping. Therefore, the denominator for AIT (n = 152) differed from that of the total study population (n = 231).

### Association of mutation status with clinicopathological characteristics and AIT

The distribution of genetic mutations differed significantly in AIT. Patients without mutations or with isolated BRAF mutations were more likely to have coexisting AIT, whereas TERT and BRAF+TERT double mutations were predominantly observed in patients without AIT (p = 0.029), suggesting that aggressive mutational profiles are less frequent in the presence of AIT (Table 2).

**Table 2:** Association of the mutation status with clinicopathological characteristics and autoimmune thyroiditis.

Variables		BRAF, N (%)	TERT, N (%)	BRAF+TERT, N (%)	No mutation, N (%)	p-value
		n varies	n varies	n varies	n varies	
**Age, years** **Mean ± SD**		48.94 ± 14.02	55.75 ± 16.80	56.73 ± 14.49	40.82 ± 16.25	**<0.001**
**Sex**	**Female**	111(82.2)	2 (50)	7 (63.6)	54 (88.5)	**0.061**
**Tumor size, mm** **Mean ± SD**		22.45 ± 14.87	37 ± 11.52	28.82 ± 15.37	25.70 ± 16.01	**0.021**
**LN metastasis**	**Yes**	27 (24.5)	2 (50.0)	5 (71.4)	18 (32.1)	**0.041**
**Stage**	**II-IV**	9 (8.20)	1 (25)	4 (57.1)	4 (7.10)	**<0.000**
**AIT**	**Yes**	53 (58.9)	1 (25)	2 (18.2)	24 (63.2)	**0.029**

PTC, papillary thyroid carcinoma; mm, mm; n, total number of cases; N, number of cases; AIT, autoimmune thyroiditis, presence of Hashimoto’s thyroiditis and Graves’ disease; BRAF, BRAF^*V600E*^ irrespective of *TERT*-p mutation; *TERT*-p, *TERT*-p irrespective of BRAF^*V600E*^, BRAF+ TERT, double BRAF^*V600E*^ and *TERT*-p mutant. Percentages were calculated based on available cases for each variable. Denominators differed across variables because of missing data.

The mutation status was significantly associated with tumor aggressiveness. Lymph node metastasis and advanced tumor stages (II–IV) were markedly more common in patients with TERT and BRAF+TERT mutations, with the highest rates observed in the double mutation group. In contrast, patients without mutations or with BRAF mutations alone presented with early-stage disease. Age and tumor size also varied across the mutation groups, with patients harboring TERT-related mutations being older and having larger tumors. No significant association was observed between the mutation status and sex (Table 2). Owing to missing values, the total number of observations differed across sections. Table 2 includes separate single tables in the supplementary sections (S2 Table).

### Association between BRAF and TERT promoter mutation status and AIT subtypes

**Fig 1:**
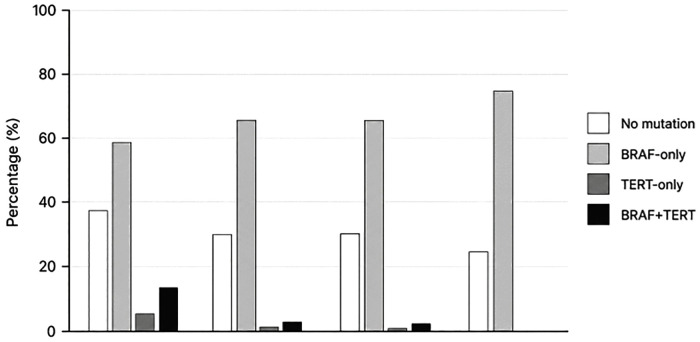
Distribution of BRAFV600E and TERT promoter mutation profiles across autoimmune thyroiditis subgroups. Relative distribution of mutation categories in patients with papillary thyroid carcinoma according to autoimmune thyroiditis subgroup. Mutation categories included no mutation, BRAFV600E-only, TERT promoter mutation-only, and coexisting BRAFV600E/TERT promoter mutations. Percentages were calculated within each subgroup. No AIT, patients without autoimmune thyroiditis; AIT, autoimmune thyroiditis; HT, Hashimoto’s thyroiditis; GD, Graves’ disease.

The distributions of BRAF and TERT promoter mutation profiles differed significantly between patients with and without AIT (P = 0.029) (Table 3). Among the patients without AIT, BRAF-only mutations were the most common (58.7%), followed by no detectable mutations (36.8%). In contrast, patients with AIT had a higher proportion of BRAF-only mutations (66.3%) and a lower frequency of TERT promoter-related alterations, particularly BRAF/TERT-p double mutations (2.5%). When the AIT subtypes were analyzed separately, similar mutation patterns were observed in patients with HT and GD. However, the association between mutation status and AIT subtype did not reach statistical significance (p = 0.163), likely due to the small number of GD cases. Overall, TERT promoter mutations, either alone or in combination with BRAF mutations, were uncommon in patients with autoimmune thyroid diseases, suggesting a less aggressive molecular profile in this group. The relative distribution of mutation categories across AIT subgroups is shown in Fig 1.

**Table 3:** Association between BRAF and TERT promoter mutation statuses and autoimmune thyroiditis subtypes.

Characteristics	No BRAF/no TERT-p	BRAF-p-only	TERT-p-only	BRAF/TERT-p	P value
**No AIT (n = 63)**	14 (36.8)	37 (58.7)	3 (4.8)	9 (14.3)	0.029
**AIT (n = 80)**	24 (30.0)	53 (66.3)	1 (1.3)	2 (2.5)	
**HT (n = 76)**	23 (30.3)	50 (65.8)	1 (1.3)	2 (2.6)	0.163
**GD (n = 4)**	1 (25)	3 (75)	0 (0.0)	0 (0.0)	

PTC, papillary thyroid carcinoma; n, number of cases; AIT, autoimmune thyroiditis, presence of both Hashimoto’s thyroiditis and Graves’ disease; HT, Hashimoto’s thyroiditis; GD, Graves’ disease; BRAF, BRAF^*V600E*^ irrespective of *TERT*-p mutation; *TERT*-p, *TERT*-p irrespective of BRAF^*V600E*^; BRAF-only, BRAF^*V600E*^ vs BRAF^*V600E*^/*TERT*-p mutant; *TERT*-p only, TERT mutant vs BRAF^*V600E*^/*TERT*-p mutant; BRAF/TERT-*p*, double BRAF^*V600E*^ and *TERT*-p mutant. Only patients with complete data on both mutation status and AITs were included in this analysis. Therefore, the total number of cases differed from that reported in Table 1. Mutation categories were mutually exclusive (BRAF-only, TERT-only, BRAF+TERT, and no mutation). Percentages were calculated within each AIT subgroup.

### Clinicopathological differences in Kazakh PTC patients based on AIT subtypes

Sex distribution, lymph node metastasis, and advanced stage (T2–T4) did not differ significantly between the patients with and without autoimmune thyroid disease (Table 4). Similar nonsignificant patterns were observed in the HT and GD subgroups. Although patients with GD showed numerically higher proportions of lymph node metastasis and advanced-stage disease, these differences were not statistically significant, likely because of the small sample size. A statistically significant difference in Ki-67 labeling index was observed between the HT and GD groups (p = 0.042), with higher values in the GD subgroup. Nevertheless, given the limited sample size of GD patients, this result should be considered exploratory. S2 Table presents a detailed comparison of clinicopathological features across the AIT subtypes (No AIT, HT, and GD). Patients with GD showed a trend toward a more aggressive tumor phenotype, with a larger tumor size and a significantly higher Ki-67 labeling index compared to the No AIT and HT groups. In contrast, clinicopathological features in patients with HT were largely comparable to those in patients without AIT. Findings in the GD subgroup should be interpreted cautiously because of the small sample size.

**Table 4:** Clinicopathological differences in Kazakh patients with PTC based on AIT subtypes.

Characteristics	Sex	P value	LN metastasis	P value	Stage	P value	Tumor Size (mm)	P value	Ki-67 LI	P value
	Female, n (%)		Yes, n (%)		T2-4, n (%)		Mean ± SD		Mean ± SD	
No AIT (n = 68)	55 (80.9)	0.991	19 (32.2)	0.328	7 (11.9)	0.37	22.91 ± 11.98	0.561	5.8 ± 6.9	0.345
AIT (n = 84)	68 (81.0)		20 (24.7)		6 (7.4)		23.64 ± 16.61		5.1 ± 5.2	
HT (n = 79)	65 (82.3)	0.47	18 (23.7)	0.328	5 (6.6)	0.405	23.3 ± 15.77	0.844	4.9 ± 4.5	0.042
GD (n = 5)	3 (60)		**2 (40)**		**1 (20)**		**29 ± 28.84**		**8.2 ± 12.4**	

PTC, papillary thyroid carcinoma; n, number of cases; AIT, autoimmune thyroiditis, presence of both Hashimoto’s thyroiditis and Graves’ disease; HT, Hashimoto’s thyroiditis; GD, Graves’ disease.

## Discussion

In this study, we comprehensively evaluated the clinicopathological and molecular characteristics of PTC in Kazakh patients, with a particular focus on the role of AIT and its subtypes HT and GD. Given the increasing incidence of AIT worldwide and its frequent coexistence with PTC, clarifying whether an autoimmune background influences tumor behavior and molecular aggressiveness remains an important clinical issue.

Another factor possibly contributing to the high prevalence of AIT in our cohort was iodine nutrition. Historically, Kazakhstan has been considered an iodine-deficient region, and iodine deficiency is a well-recognized risk factor for the development of autoimmune thyroid disorders, particularly HT. Populations transitioning from iodine deficiency to sufficiency often experience an increase in the prevalence of autoimmune thyroid disease, which may partly explain the relatively high proportion of AIT observed in our cohort compared with iodine-sufficient countries. Therefore, differences in iodine intake across regions may contribute to international variability in the reported association between AIT and PTC and should be considered when comparing data across populations.

The link between chronic inflammation and carcinogenesis is well established; however, the underlying mechanisms vary depending on the nature of the immune response and tissue microenvironment [44,45]. Autoimmune thyroid diseases are distinct immunological entities that may differentially influence thyroid tumor biology. GD is predominantly associated with a Th2-skewed immune response characterized by the production of TR antibodies, persistent thyroid stimulation, and a hyperfunctional glandular state. In contrast, HT is mainly driven by Th1-mediated immunity, which leads to chronic lymphocytic infiltration, follicular cell destruction, and progressive hypothyroidism [46].

These divergent immune profiles may create fundamentally different tumor microenvironments. In HT, sustained lymphocytic infiltration has been proposed to enhance immune surveillance, potentially restricting tumor growth and progression. This concept is supported by several studies suggesting that HT may act as a “double-edged sword,” increasing the likelihood of PTC detection, while simultaneously being associated with less aggressive tumor behavior [19,20,47]. Our findings, showing comparable clinicopathological and molecular features between HT-associated PTC and PTC without autoimmune thyroid disease, are consistent with a protective or neutral role. Patients with AIT had a tumor size, lymph node metastasis, stage distribution, and overall disease stage comparable to those observed in patients without autoimmune thyroid disease. This was further supported by the observation that a tumor size >10 mm was similarly prevalent in patients with and without AIT, as well as across the HT and GD subgroups. These results are consistent with those of previous reports, suggesting that AIT, particularly HT, does not necessarily promote aggressive tumor behavior and may coexist with PTC without adversely affecting its clinical presentation.

**Fig 2:**
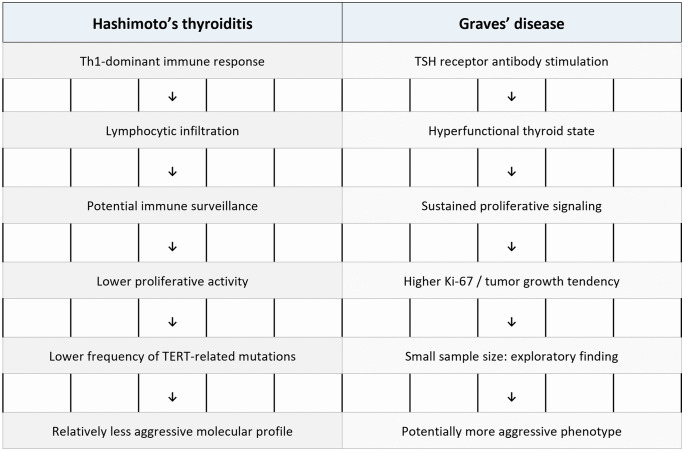
Proposed schematic model of biological differences between Hashimoto’s thyroiditis–associated and Graves’ disease–associated papillary thyroid carcinoma. Proposed biological model illustrating differences between Hashimoto’s thyroiditis (HT)-associated and Graves’ disease (GD)-associated PTC. HT-associated PTC appeared to be associated with lower proliferative activity and reduced prevalence of aggressive molecular alterations, whereas GD-associated PTC demonstrated features suggestive of increased tumor growth and proliferative potential.

Conversely, the hyperstimulatory milieu in GD may promote tumor proliferation through continuous TSH receptor activation and altered cytokine signaling, potentially facilitating tumor growth and increasing proliferative activity. Previous meta-analyses and clinical studies have reported a higher incidence and more aggressive behavior of PTC in patients with GD, although the results remain heterogeneous [21]. Consistent with these observations, our data showed that PTC cases associated with GD tended toward a more aggressive phenotype. Patients with GD showed a larger tumor size and significantly higher Ki-67 labeling index than those with HT-associated tumors, indicating increased proliferative activity. Additionally, lymph node metastasis and advanced tumor stage were more frequent in the GD subgroup, although these differences were not statistically significant. Although these findings must be interpreted cautiously given the very small number of GD cases, they are consistent with previous studies and suggest that PTC arising in the context of GD may behave more aggressively than HT-associated PTC. The hyperstimulatory autoimmune environment characteristic of GD, driven by TR antibodies and sustained immune activation, may contribute to enhanced tumor cell proliferation and growth. A schematic summary of the proposed biological differences between HT- and GD-associated PTC is presented in Fig 2. HT-associated PTC appeared to show lower proliferative activity and reduced prevalence of aggressive molecular alterations, whereas GD-associated PTC showed features suggestive of increased tumor growth and proliferative potential.

Taken together, our results highlight the heterogeneity of autoimmune thyroid diseases and their differential impacts on PTC. Rather than exerting a uniform effect, autoimmune background appears to influence PTC behavior in a subtype-dependent manner. HT is associated with a relatively indolent clinicopathological and molecular profile, whereas GD may be linked to increased proliferative activity and tumor growth; however, confirmation in larger cohorts is required.

Importantly, mutation analysis revealed a distinct relationship between the autoimmune background and molecular tumor profiles. Aggressive mutational patterns involving TERT promoter mutations, particularly BRAF/TERT-p double mutations, were predominantly observed in patients without AIT, whereas patients with AIT more frequently harbored BRAF-only mutations or lacked detectable mutations. This inverse association between AIT and TERT-related alterations suggests that autoimmune thyroid diseases may be associated with the less aggressive molecular phenotype of PTC. Given the established role of TERT promoter mutations, particularly in combination with BRAFV600E, as strong predictors of tumor aggressiveness, recurrence, and mortality, the relative scarcity of these mutations in AIT-positive patients is clinically meaningful. These findings are consistent with those of previous studies demonstrating that BRAFV600E and TERT promoter mutations, especially in combination, define the most aggressive molecular subtype of PTC [36–40]. It may partially explain the generally favorable clinicopathological profile observed in this group. TERT promoter mutations and their relative rarity in AIT-positive patients suggest that immune-mediated mechanisms counteract the emergence or clonal expansion of highly aggressive tumor subpopulations. However, given the limited number of GD cases and TERT-mutated tumors, these findings should be regarded as hypothesis-generating. To the best of our knowledge, data on the relationship between AIT and TERT promoter mutations in PTC are extremely limited, and our study is one of the first systematic analyses to address this gap.

This study had several limitations. The retrospective design and the limited number of GD-associated cases limit our ability to draw definitive conclusions about the prognostic impact of GD on PTC. Additionally, long-term outcome data, including recurrence and disease-specific survival, were not available. Nevertheless, the consistent trends observed across clinicopathological and molecular parameters suggest biologically plausible differences warranting further investigation. Furthermore, we focused on predefined, clinically relevant hotspot mutations (BRAFV600E and TERT promoter mutations (C228T and C250T)), which are well-established markers of tumor aggressiveness and are routinely used in clinical risk stratification. Although this targeted approach did not capture the full spectrum of genetic alterations, it allowed for a focused evaluation of the most clinically impactful mutations. Other genomic alterations may also contribute to tumor behavior and should be explored in future studies using broader molecular profiling approaches.

In conclusion, our findings suggest that AIT does not uniformly influence the behavior of PTC. HT appears to coexist with PTC without clear evidence of promoting aggressive clinical or molecular features, whereas different patterns were observed in GD, including increased proliferative activity and tumor growth. However, these observations should be interpreted with caution due to the limited number of GD cases. Moreover, the lower prevalence of TERT promoter-related mutations in AIT-positive patients suggests a trend toward a less aggressive molecular profile in this group, although this conclusion is based on analysis of selected hotspot mutations. These findings highlight the heterogeneity of autoimmune thyroid diseases and their potential impact on tumor behavior, warranting further investigation in larger, well-characterized cohorts with broader molecular profiling.

## Supporting information

S1 Table Comparison of autoimmune thyroid disease subtypes (AIT, HT, and GD) in Kazakh patients with PTC stratified by tumor size. (DOCX)

S2 Table Clinicopathological differences in Kazakh patients with PTC based on AIT subtypes. (DOCX)
