# Supplementary material for: Association between autoimmune thyroiditis and BRAFV600E/TERT promoter mutations in patients with papillary thyroid carcinoma from Central Asia, Kazakhstan
Source: PLoS One. 2026 Jul 20;21(7):e0351960. doi: 10.1371/journal.pone.0351960 (PMC13384275; doi:10.1371/journal.pone.0351960)
Supplement: S1 Table — (DOCX) [file pone.0351960.s001.docx]

**S1 Table. Comparison of autoimmune thyroid disease subtypes (AIT, HT, and GD) in Kazakh patients with PTC stratified by tumor size**

| Characteristics | | ≤10.0 mm |  | >10.0 mm | p-value |
| --- | --- | --- | --- | --- | --- |
|  |  | n (%) |  | n (%) |  |
| AIT | Yes | 15 (17.9) |  | 69 (82.1) | 0.99 |
| HT | Yes | 14 (17.7) |  | 65 (82.3) |  |
| GD | Yes | 1 (20.0) |  | 4 (80.0) |  |

PTC, papillary thyroid carcinoma; mm, millimeters; n, number of cases; AIT, autoimmune thyroiditis, presence of both Hashimoto's thyroiditis and Graves’ disease; HT, Hashimoto's thyroiditis; GD, Graves’ disease; Yes, patients with the history of AIT.
