# Supplementary material for: Association between autoimmune thyroiditis and BRAFV600E/TERT promoter mutations in patients with papillary thyroid carcinoma from Central Asia, Kazakhstan
Source: PLoS One. 2026 Jul 20;21(7):e0351960. doi: 10.1371/journal.pone.0351960 (PMC13384275; doi:10.1371/journal.pone.0351960)
Supplement: S2 Table — (DOCX) [file pone.0351960.s002.docx]

**S2 Table. Clinicopathological differences in Kazakh patients with PTC based on AIT subtypes**

| Characteristics | | No AIT |  | HT |  | GD |  | AIT |
| --- | --- | --- | --- | --- | --- | --- | --- | --- |
|  |  | (n=68) N (%) |  | (n=79) N (%) |  | (n=5) N (%) |  | (n=84) N (%) |
| Tumor size, mm  (Mean ± SD) | | 22.9±11.9 |  | 23.3±15.8 |  | **29±28.8*** |  | 23.6±16.6 |
| LN metastasis | Yes | 19 (27.9) |  | 18 (22.8) |  | **2 (40)** |  | 20 (23.8) |
| Stage | T2-4 | 7 (10.3) |  | 5 (6.3) |  | **1 (20)** |  | **6 (7.1) ^#^** |
| Ki-67  (Mean ± SD) | | 5.8±6.9 |  | 4.9±4.5 |  | **8.2±12.4*** |  | 5.1±5.2 |

PTC, papillary thyroid carcinoma; mm, millimeters; n, total number of cases; N, number of cases; AIT, autoimmune thyroiditis, presence of both Hashimoto's thyroiditis and Graves’ disease; HT, Hashimoto's thyroiditis; GD, Graves’ disease; Yes, patients with a history of AIT; LN, lymph node; Stage, 1 versus 2+3+4, WHO 5ed; *, the analysis was performed in No AIT and GD for comparison, with results considered significant at p < 0.05; **^#^**, the analysis was performed in No AIT and AIT for comparison, with results considered significant at p < 0.05.
